# The graded predictive pre-activation in Chinese sentence reading: evidence from eye movements

**DOI:** 10.3389/fpsyg.2023.1136488

**Published:** 2023-06-29

**Authors:** Min Chang, Kuo Zhang, Yue Sun, Sha Li, Jingxin Wang

**Affiliations:** ^1^School of Education Science, Nantong University, Nantong, China; ^2^Department of Social Psychology, Nankai University, Tianjin, China; ^3^Faculty of Psychology, Tianjin Normal University, Tianjin, China; ^4^Academy of Psychology and Behavior, Tianjin Normal University, Tianjin, China; ^5^School of Psychology, Fujian Normal University, Fuzhou, China

**Keywords:** lexical predictability, contextual constraint, graded pre-activation, Chinese reading, eye movement

## Abstract

Previous research has revealed that graded pre-activation rather than specific lexical prediction is more likely to be the mechanism for the word predictability effect in English. However, whether graded pre-activation underlies the predictability effect in Chinese reading is unknown. Accordingly, the present study tested the generality of the graded pre-activation account in Chinese reading. We manipulated the contextual constraint of sentences and the predictability of target words as independent variables. Readers’ eye movement behaviors were recorded via an eye tracker. We examined whether processing an unpredictable word in a solid constraining context incurs a prediction error cost when this unpredictable word has a predictable alternative. The results showed no cues of prediction error cost on the early eye movement measures, supported by the Bayes Factor analyses. The current research indicates that graded predictive pre-activation underlies the predictability effect in Chinese reading.

## Introduction

Prediction is a fundamental principle of language processing (Clark, 2013). Efficient language comprehension depends on two streams of information, i.e., the top-down expectation and the bottom-up conceptual input. In speech comprehension, listeners could predict the content at the end of other speakers’ turns to make efficient turn-taking using statistical regularities information in speech (Scott et al., 2009). In reading comprehension, readers could make use of contextual predictability information to facilitate word identification and semantic integration (for a review see Staub, 2015). A word’s predictability, as measured by the word’s cloze value, i.e., the proportion of participants who give this word in a non-speeded sentence completion task (Taylor, 1953), has been shown to influence reading times and saccadic behavior in reading tasks using the eye-tracking method of English, German, and Chinese (Rayner and Well, 1996; Kliegl et al., 2004; Rayner et al., 2005; Wang et al., 2010; Staub, 2015; Liu et al., 2018; Zhao et al., 2019; Chang et al., 2020a,b). Specifically, predictable words are easier to read, receive fewer and shorter fixations, and elicit longer progressive or incoming saccade length than unpredictable words, i.e., the word predictability effect. However, the mechanisms for the predictability effect in Chinese reading have not been investigated previously. Thus, the present study aims to determine how prediction occurs, i.e., the mechanism of word predictability effect in Chinese reading.

Two competing theoretical accounts explain the mechanisms for predictability effects, each of which has different predictions for processing unexpected words (Luke and Christianson, 2016; for a review see Staub, 2015). First, the word prediction could be defined as an “all-or-none” process in which readers may maintain specific, discrete predictions of upcoming perceptual input, also termed *lexical prediction* (also see Delong et al., 2014). According to this *lexical prediction account*, strong constraining sentences support expectations for predictable words with much facilitation. Reading can be facilitative when readers encounter predictable words but slow down when readers encounter unpredictable words in a sufficient constraining context, i.e., producing the prediction error cost (Kutas et al., 2011; Luke and Christianson, 2016). For example, readers would predict the most probable word *gift* in the constraining sentence “*Today was Annie’s birthday, her mother bought her a-.*” This predictable word *gift* would be processed quickly as it matches readers’ expectations. On contrary, readers might be surprised when encountering an unexpected word like *book*, then they would spend more time reading this unexpected word (i.e., prediction error cost) as they must suppress the activated *gift.* While a neutral constraining sentence like “When Annie went home, her mother brought her a-” provides little contextual information to readers. Thus, processing the unpredictable words would rarely incur prediction error cost as no predictable word is pre-activated. Therefore, according to the *lexical prediction account*, the comparison of processing unpredictable words between the constraining context and the neutral context would cause a prediction error cost.

Second, prediction in language comprehension could also involve *graded pre-activation* so readers make diffuse, cost-free, and ubiquitous pre-activation of likely upcoming input (Luke and Christianson, 2016; for reviews, see Staub, 2015; Kuperberg and Jaeger, 2016). Compared to the *lexical prediction account*, the key prediction of this account is that processing the unpredictable word would not incur a prediction error cost when the expected word is another more possible alternative in a strong constraining sentence. Because not only the predictable word but also the unpredictable word would be pre-activated before the perceptual input is encountered. In the neutral context of the above example, readers would pre-activate a set of words that suit the context, like *book*, *hat*, *skirt*, and *guitar*. Please notice that these words mentioned above are nouns, which could be pre-activated at syntactic or semantic representation even if the word identities are not. Readers may not be able to predict *gift*, but they can be confident that the upcoming word will be a noun or something that could be carried. Thus, even if people do not predict specific words, they could predict some aspects of future stimuli (Pickering and Gambi, 2018). Therefore, according to the *graded pre-activation account*, the comparison of processing unpredictable words between the constraining context and the neutral context would not cause a prediction error cost.

The graded pre-activation account has been well-demonstrated in English reading (for a review see Kuperberg and Jaeger, 2016), as evidenced by the reliable correlation between word predictability (measured as word surprizal or cloze probability) and processing times (Monsalve et al., 2012; Smith and Levy, 2013; Goodkind and Bicknell, 2018), N400 amplitude (Delong et al., 2005; Frank et al., 2015), or neural activity (Henderson et al., 2016). Specifically, the word predictability was inversely correlated with reading times (e.g., gaze duration in Goodkind and Bicknell, 2018), N400 amplitudes of words (Delong et al., 2005), and changes in brain activation levels in the temporal, parietal, occipital, cingulate, and frontal regions (Carter et al., 2019). In addition, Luke and Christianson (2016) conducted a large-scale survey that provided cloze values for words in the Provo Corpus. Their results showed that most words had a more-expected competitor but with no misprediction error cost. Even if the word identity was rarely predicted, its semantic and morphosyntactic information was predictable. These findings support the *graded prediction account* but not the specific *lexical prediction account*. The null prediction error cost (as the key opinion of graded pre-activation account) also has been demonstrated by Frisson et al. (2017) using a controlled-experimental design with an eye-tracking method using a corpus study with high ecological validity.

Frisson et al. (2017) jointly manipulated the contextual constraint of sentences and the cloze probability of target words to explore the cognitive mechanism of predictability effects in English. They compared the processing of the same unpredictable word (e.g., *chair*) in the constraining context (e.g., “The young nervous paratrooper jumped out of the *plane/chair* when he heard the shots”) and the neutral context (e.g., “The tired movie maker was sleeping in the *plane/chair* when he was woken up by a scream”) to test the prediction error cost. Also, the cloze values for unpredictable words in the constraining and neutral sentences were comparable. Their results showed significant word predictability effects and contextual constraint effects, but null prediction error cost in the early or later eye movement measures. This study firstly provided evidence from the controlled experimental design for the absence of a prediction error cost and further supported that the *graded pre-activation* but not the *lexical prediction account* underlies the mechanism of word predictability effects.

Notably, the null prediction error cost in constraining sentences might be due to the priming effect from the pre-target word area. The richer information preceding the target words might facilitate automatic priming to the target words in the strong constraining sentences but not the neutral sentences (see Kuperberg and Jaeger, 2016). Although whether there is an interference from the priming effect in predictive processing is unclear, it is recommendable to control the pre-target region to investigate the predictive processing, especially in Chinese such visually denser scripts.

For Chinese reading, there have been several studies investigating how the word predictability affects eye movement behaviors or interplays with other linguistic factors (Rayner et al., 2005; Wang et al., 2010; Liu et al., 2018; Zhao et al., 2019; Chang et al., 2020a,b). However, studies of Chinese to date have yet to investigate the mechanism of word predictability effects. Whether prediction error cost exists in Chinese reading is still being determined. Chinese scripts lack morphosyntactic information, which readers use as cues for prediction. Moreover, parafoveal processing is more efficient in Chinese than English (Vasilev and Angele, 2017). Thus, readers might heavily rely on bottom-up perceptual processing in Chinese reading. Such Chinese script characteristics might make it hard to produce a specific word prediction in Chinese reading. Therefore, predictive processing might rely on graded pre-activation rather than lexical prediction. The present study aimed to provide experimental evidence for the *graded pre-activation account* in Chinese reading.

Accordingly, the present study was a follow-up to a previous study (Frisson et al., 2017) but further made more rigid control of the pre-target context. There is no explicit visual marker in Chinese to demarcate work boundaries (Li et al., 2015). Characters, the component of words, are created from differing numbers of strokes. These characteristics, therefore, bring about the increased visual density in this language and lead to deeper parafoveal pre-processing, as demonstrated by the well-established semantic preview effect in Chinese, which is equivocal in English (Zhou et al., 2013; Rayner et al., 2014). The different content immediately before the target words might influence the processing of target words differently (Reichle et al., 2003). Moreover, early eye-tracking studies have found that transitional probabilities (i.e., the statistical likelihood that word N will follow word N-1) between word N-1 and word N influence fixation times on word N (McDonald and Shillcock, 2003; Frisson et al., 2005; Wang et al., 2010). Hence, it is necessary to control the influence of the pre-target region across conditions.

Given the above considerations, we manipulated the contextual constraint and word predictability to address the question using a natural sentence reading task, consistent with Frisson et al. (2017). However, we went further by constructing compound sentences, with the first half-sentences controlling contextual constraint and the second half-sentences having identical content at least three characters before the target words to control the possible priming effect or pre-target influence on the target words. We obtained the contextual constraint effect, word predictability effect, and the prediction error cost by three comparisons: (1) constraining context-unpredictable (CU) vs. constraining context-predictable (CP), testing the word predictability effect; (2) neutral context–predictable (NP) vs. constraining context-predictable (CP), testing the contextual constraining effect, and (3) constraining context-unpredictable (CU) vs. neutral context-unpredictable word (NU), testing the prediction error cost. According to the *lexical prediction account*, unpredictable word processing in the constraining context would result in extra prediction error cost but not in the neutral context. Thus, we compared CU and NU to evaluate the prediction error cost, as Frisson et al. (2017).

We expected to find the typical word predictability effect, i.e., predictable words yielding shorter reading times than unpredictable words. We also expected the significant contextual constraint effect, i.e., the strong constraining sentences but not the neutral sentences make target words read faster. The contextual effects and the standard word predictability effects in the first-pass reading measures demonstrated that we manipulated the two factors successfully. However, the two effects mentioned above are not key evidences to our hypothesis. The prediction error cost (CU vs. NU) is the primary evidence for distinguishing the two accounts. Specifically, if readers spent longer time on reading unpredictable word in CU than in NU (i.e., significant prediction error cost), then the result supported the *lexical prediction account*, otherwise (null prediction error cost) *supported the graded pre-activation account*.

## Materials and methods

### Ethics approval

The study was approved by the research ethics committee at the Tianjin Normal University and conducted according to the Declaration of Helsinki principles.

### Participant

Forty-four undergraduates aged 18–26 years (*M* = 20.5 years, 34 female) from the author’s university participated in the eye-tracking experiment for remuneration. The participant number was the same as Frisson et al. (2017). All were native Chinese readers, screened for normal acuity (more excellent than 20/40 in Snellen values) using a Tumbling E eye chart (Taylor, 1978), and naive to the purpose of the experiment. Informed consent was obtained from all individual participants in the study.

### Design and stimuli

We constructed 48 sets of sentence frames, a number larger than Frisson et al. (2017). The experiment used a within-subjects design with the factors of sentence constraint (Constraining, Neutral) and word predictability (Predictable, Unpredictable) as independent variables. See Table 1, each sentence frame had a strong constraining sentence and a neutral sentence. The first half-sentence was manipulated to control the contextual constraint; predictable or unpredictable target words were inserted in the middle of the second half-sentence. At least three characters before target words were identical in the constraining and neutral conditions (excluding only five sets of sentences). As stated in the introduction, we conducted three comparisons to obtain the contextual constraint effect, word predictability effect, and prediction error cost. The most crucial comparison was the third one, i.e., constraining context-unpredictable word (CU) vs. neutral context-unpredictable word (NU), testing the prediction error cost. The significant prediction error cost indicates that an unexpected word in a constraining context with a predictable alternative will incur a processing cost, which supports the *lexical prediction account*.

**TABLE 1:** An example stimulus.

Condition	The first half-sentence	The second half-sentence
Constraining context–predictable (CP)	T台上的刘雯小姐优雅地款款走来,	这位走向世界的中国 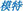 让外国友人看到了东方之美。
Constraining context-unpredictable (CU)	T台上的刘雯小姐优雅地款款走来，	这位走向世界的中国 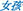 让外国友人看到了东方之美。
Neutral context-predictable (NP)	舞台上的邓琦小姐散发着优雅知性的气质，	这位走向世界的中国 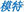 让外国友人看到了东方之美。
Neutral context -unpredictable (NU)	舞台上的邓琦小姐散发着优雅知性的气质，	这位走向世界的中国 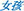 让外国友人看到了东方之美。

Target words are shown in bold. The constraining sentence translates as “Miss Liu Wen on the runway comes gracefully, and this Chinese 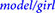 who is famous around the world shows foreign friends the beauty of the East.” The neutral sentence translates as “Miss Deng Qi on the stage exudes an elegant and intellectual temperament; this Chinese 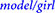 who is famous around the world shows foreign friends the beauty of the East.” Please note that the target word, such as 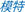
(model) in the first condition of neutral context, was the same as in the CP condition. Thus we labeled it as NP. The NP and NU did not differ in predictability, word frequency, and complexity.

In the cloze test, students were given the sentences truncated immediately before the target word and asked to provide the next word in the sentences. Twenty-two college students who did not participate in the experiment completed the cloze test. A predictable or unpredictable word was embedded in the constraining context (labeled CP and CU, respectively, see Table 1). The same two words were embedded in the corresponding neutral context and embedded in the constraining context. Given that the two target words, such as model/girl in the neutral context, were the same as targets in the constraining context, we labeled them as NP and NU, following Frisson et al. (2017). Please note that NP and NU were unpredictable because the neutral context did not provide strong word constraints. The mean cloze probability of the target words in the four conditions (CP, CU, NP, and NU) were 0.75 (*SD* = 0.16), 0.02 (*SD* = 0.04), 0.05 (*SD* = 0.06), and 0.04 (*SD* = 0.08), respectively. In the constraining context, *t*-tests showed that the cloze values for CP were significantly higher than for CU [*t*(94) = 30, *p* < 0.001]. In the neutral context, the two unpredictable targets had comparable cloze values [*t*(94) = 1.07, *p* = 0.288]. Importantly, the cloze values for the same unpredictable word (such as *girl*) in constraining and neutral contexts were comparable [*t*(94) = 1.52, *p* = 0.13].

The two target words in one sentence frame were matched for word frequency [ Cai and Brysbaert, 2010; Predictable: *M* = 64/million, *SD* = 80; Unpredictable: *M* = 44/million, *SD* = 104; *t*(94) = 1.06, *p* = 0.291] and the whole word complexity in strokes [Predictable: *M* = 17.41, *SD* = 5.11; Unpredictable: *M* = 15.88, *SD* = 4.97; *t*(94) = 1.50, *p* = 0.137]. Forty participants evaluated sentences naturalness (using a 7-point scale, ranging from 1 = entirely unnatural to 7 = entirely natural). The average ratings were 5.41 (*SD* = 0.74), 5.31 (*SD* = 0.71), 5.32 (*SD* = 0.66), and 5.20 (*SD* = 0.7) for each conditions, respectively. The ANOVA analysis showed that the four conditions were comparable in naturalness [*F*_(3, 188)_ = 0.85, *p* = 0.468].

We adopted a counterbalanced design in which the experimental sentences were divided into four lists, and one version of each sentence frame was in one list. Each participant read one list with equal numbers of sentences in each condition. Each list also included 40 filler sentences and began with six practice sentences. Eleven participants were randomly allocated to each list.

### Apparatus and procedure

An SR Eyelink 1000 plus eye tracker tracked right-eye movements during binocular viewing at 1000 Hz. Stimuli were displayed in Song 32-point font as black-on-white text on a high-resolution (1920 × 1080 pixels) monitor with a fresh rate of 60 Hz. At 65 cm viewing distance, each character subtended 1° and so was of normal size for reading.

Participant took part individually and was instructed to read normally and for comprehension. At the start of the experiment, a 3-point horizontal calibration procedure was performed across the same line as each sentence presentation (ensuring 0.30° or better spatial accuracy for all participants). Calibration accuracy was checked before each trial and the eye-tracker recalibrated as required to maintain high spatial accuracy. At the start of each trial, a fixation square equal in size to one character was presented on the left side of the screen. Once the participant fixated on this location, the first half-sentence was presented with the first character replacing the square. Participant pressed the space key once they finished reading the first half-sentence. Then the same fixation square was presented again at the same position and disappeared once the participant fixated it, then the second half-sentence was presented. Participant pressed a response key once they finished reading the second half-sentence. This was replaced by a comprehension question requiring a yes/no button-press response on 25% of trials. The experiment lasted approximately 30 min for each participant.

### Data analysis

Accuracy for answering comprehension questions was high for all participants (*M* = 84%, *SD* = 0.06, range = [73%, 95%]). We output the data of the second half-sentences and thus removed the data based on the second half-sentences. Following standard procedures, short (< 80 ms) and long (> 1200 ms) fixations were removed. Trials with head-movement, tracking-loss, or error were excluded, which affected seven trials (0.3%), as were trials for sentences receiving fewer than six fixations, which affected 99 trials (4.7%). In total, 5% of trials (106) were removed. The remaining data were analyzed by linear mixed-effects models (LMEs; Baayen et al., 2008) for continuous variables and generalized mixed-effects models for binomial variables, using the lme4 package (Version 1.1-21; Bates et al., 2015) in R (R Development Core Team, 2016). For all measures, models with the maximum random-effects structure were used (Barr et al., 2013), with the three comparisons as fixed factors and participant and stimuli as crossed random effects. If models did not converge, the random-effects structure was reduced by first trimming this for stimuli. Log-transformed fixation-time effects are reported alongside untransformed means. Following convention, *t*/*z* values > 1.96 were considered significant.

## Results

We expected significant word predictability effects and contextual constraint effects on the early eye movement measures and explored whether unpredictable words in constraining sentences incur processing costs on early word identification or later semantic integration. Thus, consistent with Frisson et al. (2017), we reported four measures of first-pass reading for the target words, i.e., the word-skipping (SKIP, probability of not fixating a word during first-pass reading), first-fixation duration (FFD, duration of the first fixation on a word during first-pass reading), single-fixation duration (SFD, duration of the first fixation on a word receiving only one first pass fixation), gaze duration (GD, sum of all first pass fixations on a word). We also reported three measures concerning later semantic integration, i.e., regressions-out rate (RO, probability of first-pass regression from a word), regression path duration (RPD, the sum of all fixation durations beginning with the initial fixation on the target word and ending when the eyes exited the word to the right, including time spent rereading earlier words and time spent rereading the word itself) and total reading time (TRT, sum of all fixations on a target word). Target word means were shown in Table 2, and statistical effects were summarized in Table 3.

**TABLE 2:** Means and standard errors for target word measures (*M* ± *SE*).

Measures	Constraining		Neutral	
	Predictable	Unpredictable	Predictable	Unpredictable
Skipping (%)	31 (2)	27 (2)	25 (2)	24 (2)
FFD (ms)	236 (5)	250 (5)	237 (4)	249 (5)
SFD (ms)	235 (5)	246 (5)	238 (4)	245 (5)
GD (ms)	251 (6)	272 (7)	264 (6)	274 (7)
RPD (ms)	307 (14)	333 (12)	307 (11)	361 (16)
RO (%)	10 (2)	14 (2)	11 (2)	16 (2)
TRT (ms)	348 (11)	371 (11)	351 (10)	384 (12)

**TABLE 3:** Summary of statistical effects (continuous variables were log-transformed).

Measures	Comparison	*b*	CI	*SE*	*t/z*	*p*
SKIP	Intercept	-1.1	[−1.36, −0.86]	0.12	−8.93	<0.001
	Predictability	-0.2	[−0.48, 0.09]	0.15	−1.34	0.182
	Constraint	-0.34	[−0.63, −0.05]	0.15	−2.3	0.022*
	Prediction error cost	-0.19	[−0.49, 0.10]	0.15	−1.29	0.198
FFD	Intercept	5.43	[5.38, 5.47]	0.02	236.02	<0.001
	Predictability	0.06	[0.02, 0.11]	0.02	2.63	0.009*
	Constraint	0.02	[−0.03,0.06]	0.02	0.73	0.464
	Prediction error cost	-0.02	[−0.06,0.03]	0.02	−0.68	0.494
SFD	Intercept	5.42	[5.37, 5.47]	0.02	228.39	<0.001
	Predictability	0.05	[0.01, 0.10]	0.02	2.2	0.028*
	Constraint	0.03	[−0.02, 0.08]	0.02	1.32	0.187
	Prediction error cost	-0.01	[−0.06, 0.03]	0.02	−0.58	0.565
GD	Intercept	5.48	[5.43, 5.54]	0.03	209.57	<0.001
	Predictability	0.08	[0.03, 0.13]	0.03	2.89	0.004*
	Constraint	0.06	[0.01, 0.11]	0.03	2.16	0.031*
	Prediction error cost	-0.01	[−0.06, 0.04]	0.03	−0.26	0.793
RPD	Intercept	5.61	[5.54, 5.68]	0.03	169.17	<0.001
	Predictability	0.11	[0.04, 0.19]	0.04	3.11	0.002*
	Constraint	0.05	[−0.02,0.12]	0.04	1.28	0.202
	Prediction error cost	0.03	[−0.04, 0.10]	0.04	0.94	0.346
RO	Intercept	-2.05	[−2.32, −1.81]	0.12	−16.37	<0.001
	Predictability	0.44	[−0.03, 0.92]	0.24	1.85	0.064
	Constraint	0.15	[−0.34, 0.64]	0.25	0.6	0.546
	Prediction error cost	0.18	[−0.22, 0.60]	0.21	0.89	0.372
TRT	Intercept	5.72	[5.66, 5.79]	0.03	171.51	<0.001
	Predictability	0.05	[−0.01, 0.12]	0.04	1.55	0.121
	Constraint	0.02	[−0.05, 0.09]	0.03	0.67	0.506
	Prediction error cost	0.03	[−0.04, 0.09]	0.03	0.76	0.448

Asterisks indicate significant effects where *t/z* > 1.96. CI = 95% confidence Interval.

### Word predictability effect and contextual constraining effect

**FIGURE 1:**
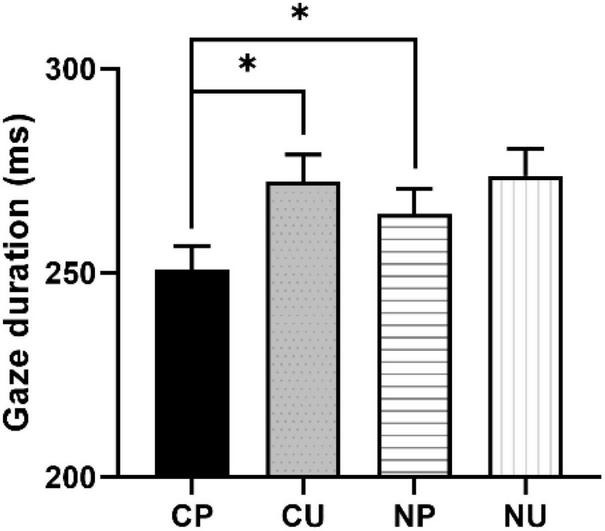
Context-predictable (CP), CU, NP, and NU represent constraining context with predictable word, constraining context with unpredictable word, neutral context with predictable word, and neutral context with unpredictable word, respectively. The contrast between CP and NP represents the contextual constraining effect; the contrast between CP and CU represents the word predictability effect; the contrast between CU and NU represents the prediction error cost. Figure describes the gaze duration in each condition. Asterisks indicate significant effect where *t* > 1.96.

We observed significant word predictability effects (CP vs. CU) and contextual constraining effects (CP vs. NP) on the first pass reading measures (see Figure 1). The word predictability effects, significant on FFD, SFD, and GD, were due to longer reading times for CU than CP conditions (FFD: *b* = 0.06, CI = [0.02, 0.11], *SE* = 0.02, *t* = 2.63; SFD: *b* = 0.05, CI = [0.01, 0.1], *SE* = 0.02, *t* = 2.2; GD: *b* = 0.08, CI = [0.03, 0.13], *SE* = 0.03, *t* = 2.89).^1^ The comparison between CP and NP revealed significant contextual constraining effects on the early skipping rate (*b* = −0.34, CI = [−0.63, −0.05], *SE* = 0.15, *z* = −2.3) and gaze duration (*b* = 0.06, CI = [0.01, 0.11], *SE* = 0.03, *t* = 2.16). Readers made more skipping and shorter first-pass fixation durations on the target word. The clear word predictability and contextual constraining effects indicated that we manipulated the two factors successfully.

### Prediction error cost

Most crucially, the prediction error cost was not significant on all the measures (| *z*/*t| s* < 1.3), i.e., an unexpected word did not incur processing cost in the constraining context with a predictable alternative, compared to the same target word in the neutral context.

We conducted Bayes factors analyses (Kass and Raftery, 1995) to determine the strength of the evidence for the null prediction error cost on the first-pass fixation time measures. The analyses were conducted using the lmBF function within the BayesFactor package (Version 0.9.12-4.2; Morey et al., 2015; R Development Core Team, 2016). Analyses were conducted with scaling factor for g-priors set to 0.5, using 10,000 Monte Carlo iterations. We first computed the Bayes Factor for a model with a fixed effect of prediction error cost (CU vs. NU) and random participant and item intercepts of FFD, SFD, and GD, i.e., BF_1_. Then we computed Bayes Factor for a model with only random participant and item intercepts, i.e., BF_0_. The critical value was the ratio of BF_1_ and BF_0_, i.e., BF_10_, it is itself a Bayes Factor comparing the model with an effect of prediction error cost and participant and item intercepts, to a model with the only participant and item intercepts. According to Vandekerckhove et al. (2015), Bayes Factors (BF_10_ < 1/3) were taken to provide moderate to strong evidence for the null model. Thus, the present results (FFD, BF_10_ = 0.11; SFD, BF_10_ = 0.03; GD, BF_10_ = 0.27) provided moderate to strong evidence for the null model, i.e., the null prediction error cost.

## Discussion

In the present experiment, we manipulated the contextual constraint of sentences and word predictability to investigate whether there is a prediction error cost in Chinese reading. We tested the prediction error cost by comparing the processing of unpredictable words between constraining contexts and neutral contexts (i.e., CU vs. NU). The results showed significant contextual effects and standard word predictability effects in the early stage of word processing, with shorter reading times (FFD, SFD, and GD) for more predictable words, which is in line with previous findings from Chinese studies (Rayner et al., 2005; Wang et al., 2010; Liu et al., 2018; Zhao et al., 2019; Chang et al., 2020a,b). Importantly, no significant prediction error cost was observed across a wide range of eye movements, i.e., the reading is not disruptive if the readers encounter the unpredictable word in a strong constraining sentence with a predictable alternative, supported by the Bayes factor analyses. This result resonated with findings from English studies (Frisson et al., 2005, 2017; Luke and Christianson, 2016). In particular, the findings suggested that readers make diffuse and graded pre-activation of likely upcoming input.

The current experiment adopted a similar design as Frisson et al. (2017). The key comparison between unpredictable words in the constraining and neutral sentences showed no prediction error cost on the fixation duration measures both for Frisson et al. and the present study. This is what we and Frisson et al. (2017) have found in common, indicating that the *lexical prediction account* would not seem able to account for the predictability effect both in English and Chinese. Notably, the present study differed from Frisson et al. (2017) on the numerical trend. They found a numerical trend in the opposite direction, i.e., the processing advantage for unpredictable words in constraining sentences compared to neutral sentences. Although this processing benefit did not reach significance on reading time measures, this trend was significant in the first pass regression rate (*z* = −2.03). The significant benefit of unpredictable words in constraining sentences might be due to the semantic priming effect or the transitional probability effect, i.e., the statistical likelihood that a word preceding the target might influence target word processing.

Like Frisson et al. (2017) study, the present study provided clear and strong evidence for null prediction error cost (*t/z* < 1.29). Unlike Frisson et al. (2017) we did not find significant benefits for unpredictable words in constraining sentences when controlling the pre-target region, providing stronger support for *graded pre-activation account*. The characteristics of the Chinese language could explain this. Chinese lacks overt cues (markers for number, gender, the tense of verbs, and case) to syntactic structure, which a reader utilizes to produce predictions about upcoming stimuli in English (see Kuperberg and Jaeger, 2016 for a review). Furthermore, the word predictability is lower in Chinese than in English, as shown by the comparison between cloze probability reported by Pan et al. (2021) in Beijing Sentence Corpus (BSC) and that by Luke and Christianson (2016) in Provo Corpus. The grand mean of cloze scores for the words in BSC is 0.07, far less than that reported in Luke and Christianson (*M* = 0.13). Thus, the sentence constraint in Chinese may be weaker than that in English. It is reasonable that we found more consistent results on the several eye movement measures.

The findings are consistent with the multi-representational hierarchical generative architecture, which views prediction as a graded and probabilistic phenomenon (Kuperberg and Jaeger, 2016). Also, this architecture suggests distinguishing between predictive pre-activation and pre-activation through priming. The present study attempted to control interference from the priming effect across conditions by constructing compound sentences in which the first half-sentences controlled the contextual constraint and the second half-sentences were identical at least three characters before the target words. Thus, the content of the pre-target region was identical in the constraining and neutral sentences. The null prediction error cost on the first pass reading measures and the later eye movement measures suggest that encountering an unexpected word in a constraining sentence does not interrupt early lexical identification and later semantic integration. Readers pre-activate not only one specific item but a range of possible words. The present study confirmed the graded pre-activation mechanism of predictive processing in Chinese reading.

### Limitations and future directions

The study had one limitation. The number of participants in the cloze task might influence the cloze value of words. There is a positive correlation between the number of participants and the precision of word’s cloze value. Our present study recruited 22 participants for the cloze task. Although we successfully balanced the cloze values between CU and NU, however, the sample size might be not big enough provide a precise cloze value of a word.

Thus, future studies could recruit as many participants as possible to obtain more precise word cloze value. Besides, cross-linguistic studies are highly needed to explore how linguistic characteristics (e.g., word space, word length, and complexity) influence predictive language processing. In addition, to improve the external validity, studies about predictive language comprehension of special readers (e.g., non-native speakers, children with dyslexia, and older adults) are needed. These studies will inform us of the mechanism of reading difficulty for non-native speakers, children with dyslexia, and older adults.

## Conclusion

In summary, we conducted an eye-tracking experiment to investigate whether processing an unpredictable word incurs prediction error cost when there is a predictable alternative. The null prediction error cost supports that the graded pre-activation account underlies the word predictability effect in Chinese reading.

## Data availability statement

The original contributions presented in this study are included in the article/supplementary material, further inquiries can be directed to the corresponding author.

## Ethics statement

The studies involving human participants were reviewed and approved by the research Ethics Committee at Tianjin Normal University and conducted according to the Declaration of Helsinki principles. The patients/participants provided their written informed consent to participate in this study.

## Author contributions

MC and JW designed the experiment and wrote the manuscript. MC and YS experimented and analyzed the data. KZ and SL provided good suggestions. All authors contributed to the article and approved the submitted version.

## Footnotes

1 The word predictability effect was also significant on RPD while we did not mention it in the Results and Discussion. As the results on RPD, RO, and TRT might represent a mixture of predictability effect and semantic integrative effect. We want to obtain the clear and genuine predictability effect. Following the tradition of eye movement research, however, we reported these later eye movement measures in the table which could be accessible for other researchers for meta-analysis. Thus, we did not mention and discuss these later eye movement measures in sections “Results and Discussion.”
